# COVID-19 pandemic and mental health of a sample of Brazilian caregivers of people with dementia

**DOI:** 10.1590/1980-57642021dn15-040004

**Published:** 2021

**Authors:** Andréia Schmidt, Maryam Furlan Ayoub, Yara Luana Pereira de Souza, Ana Tereza Bittencourt Guimarães, Maria Paula Foss

**Affiliations:** 1 Psychology Department, Universidade de São Paulo – Ribeirão Preto, SP, Brazil.; 2 Postgraduate Program in Biosciences and Health, Universidade Estadual do Oeste do Paraná – Cascavel, PR, Brazil.

**Keywords:** caregivers, dementia, stress, quarantine, COVID-19, cuidadores, demência, estresse, quarentena, COVID-19

## Abstract

Social distancing policies adopted in the COVID-19 pandemic generated a negative psychological impact on the general population and can affect caregivers of people with dementia more severely.

**Objective:**

This study screened for indications of mental health problems among informal caregivers of people with dementia. Main changes in the people’s routine and behavior resulting from the social isolation measures adopted due to the pandemic were also investigated.

**Method:**

Thirty-five informal caregivers of people with dementia from a medium-sized Brazilian city responded to a telephone interview.

**Results:**

Risks for mental health problems were found in 31.4% of the sample. These participants stated that they found it very difficult to deal with routine care changes during the pandemic. The variables related to the caregiver’s characteristics and those related to changes in routine significantly affected the caregiver’s mental health scores.

**Discussion:**

Indices of mental disorders in the studied sample did not differ from the prevalence of mental health problems in the general population during COVID-19 pandemic; however, participants reported worsening symptoms such as nervousness, sadness, and sleep during quarantine.

**Conclusions:**

Results show the complexity of this topic and the need for individual care for this group, especially in situations like the COVID-19 pandemic.

## RESUMO

As medidas de isolamento social adotadas na pandemia de COVID-19 geraram um impacto psicológico negativo na população em geral e podem afetar mais duramente cuidadores de pessoas com demência.

**Objetivos:**

Esta pesquisa rastreou indicativos de problemas de saúde mental entre cuidadores informais de pacientes com demência. As principais modificações de rotina e comportamento dos pacientes decorrentes das medidas de isolamento social adotadas em função da pandemia também foram investigadas.

**Métodos:**

Trinta e cinco cuidadoras informais de pessoas com demência de uma cidade brasileira de médio porte responderam a uma entrevista por telefone.

**Resultados:**

Riscos de problemas de saúde mental foram encontrados em 31,4% da amostra. Esses participantes afirmaram encontrar muitas dificuldades para lidar com as mudanças de rotina de cuidados durante a pandemia. As variáveis relativas às características do cuidador e as relativas às alterações de rotina afetaram significativamente os escores de saúde mental do cuidador.

**Discussão:**

Os índices de indicadores de risco para problemas mentais na amostra não diferiram da população em geral durante a pandemia de COVID-19, mas os participantes relataram piora de sintomas como nervosismo, tristeza e sono durante a quarentena.

**Conclusões:**

Os resultados apresentados mostram a complexidade do tema e a necessidade de cuidados especiais para cuidadores de pessoas com demência, especialmente em situações como a pandemia de COVID-19.

## INTRODUCTION

The severe acute respiratory syndrome coronavirus 2 (SARS-CoV-2) pandemic has led countries around the world to adopt social distancing measures to control COVID-19 cases.^1^ These social distancing measures can have various negative emotional consequences for individuals, such as increased anxiety, anger, depression, agitation, and stress.^1^ A review^2^ of the psychological effects of quarantine periods on individuals in different epidemic situations (Ebola in Africa and H1N1 and SARS in different countries) found that fear, frustration, and boredom were common symptoms that, when prolonged, can lead to posttraumatic stress, anger, and avoidance behaviors. The lack of necessary supplies was associated with anxiety and frustration, while inadequate information led people to fear the worst or have difficulties adhering to the required behaviors.^2^ Some authors have highlighted that the characteristics of the disease affect differentially socioeconomic groups due to inequality and thus exacerbate their effects. In this sense, COVID-19 may be considered a syndemic,^3^ since it affects individuals differently, either due to preexisting comorbid conditions (such as obesity or diabetes) or due to social conditions, thus allowing or limiting access to quality health care or the requirement to socially isolate. The concept of syndemic is important because it allows the recognition that political and social factors can promote, perpetuate, or exacerbate the effects of COVID-19.^4^

Individuals belonging to risk groups, such as older adults and adults with comorbidities, have been most severely affected by COVID-19.^5,6,7^ The suffering may worsen in groups with unfavorable psychological conditions even before the quarantine, such as in people with dementia, a condition classified as a major neurocognitive disorder (MND).^8^ (In this study, the terms *dementia* and *MND* are used interchangeably.) This group does not understand the need for health and social isolation measures for themselves and their caregivers. In Brazil and other low- and middle-income countries, the majority of caregivers responsible for people with dementia are women, with some degree of kinship with the patient (informal caregivers) and over 50 years of age.^9^ Taking care of a family member diagnosed with dementia without any specific preparation for this can result in high stress and burden.^10,11,12^ The provision of continuous care to older adults regarding daily living activities and the management of problem behaviors has been considered the most strenuous task for caregivers.^11^

Behavioral changes in older adults with MND may be more frequent during social distancing situations, which probably increases these caregivers’ stress and burden. The burden may increase, given changes in the family member’s routine, which include a series of protective measures and having to deal with the consequences of the social distancing itself. However, the impact of the pandemic on caregivers of people with MND is unknown. Most of the studies focus on the impact of isolation on the general population^1^ or older adults in a general way.^13^ Assessing caregivers’ psychological status and the difficulties that most affect them can help outline support measures, which will impact their well-being and that of the people with MND.

This study aimed to (a) screen for indications of mental health problems among informal caregivers of people with dementia, (b) investigate the main modifications of the routine and the difficulties faced by these caregivers in managing the changes during the quarantine period, and (c) investigate the perception of these caregivers regarding possible changes in the behavior of the person with MND during this period. Based on these data, we investigated whether there is a correlation between the risk indicators for mental health problems presented by the caregivers and the changes in the routine, specific characteristics of the caregivers’ lives and characteristics of the people with MND.

## METHODS

### Participants

**Figure 1.**
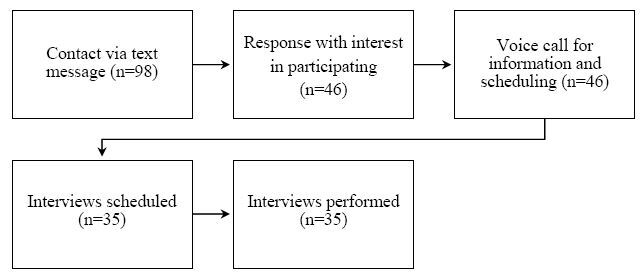
Flowchart of the recruitment process of the participants.

Participants were 35 informal caregivers (all women) of family members diagnosed with dementia, aged between 21 and 73, living in a medium-sized Brazilian city. The inclusion criteria were primary caregiver of a person with dementia, not receiving remuneration for the care, and providing care at least 20 hours of care per week. The exclusion criteria were being under 18 years of age, not being the main caregiver and/or spending less than 20 hours a week with the person with dementia, and receiving a salary to be the caregiver. The sample was recruited from a list of people interested in participating in support groups for informal caregivers of people with dementia. This list had the phone numbers of people who cared for people with dementia. Ninety-eight people were contacted via the mobile phone messaging application “WhatsApp,” 35 (35.7%) of whom agreed to be interviewed. Figure 1 shows the recruitment flow of the participants.

We assumed a large-effect size, considering the homogeneity of the target population (TE=0.9). A sample size of 38 participants was required in order to ensure a power of 0.80 and type I error of 0.05, assuming a t distribution. Due to the withdrawal of some participants, we obtained an n=35, which led to a power analysis between 0.78 and 0.84, considered sufficient to answer the hypotheses.

### Instruments

#### Interview script

Produced by the authors, the interview had 42 questions (mostly close-ended) that investigate:

- Caregiver and care receiver characteristics – 19 questions (e.g., age, occupation, education, income, housing conditions).
- Information about the care routine during the quarantine – 11 questions. Questions about fear of contamination and changes in general and care routine during quarantine were assessed on a Likert scale (1–5, ranging from nothing/not at all to extremely). Questions about implementation of hygiene and social distance measures were also assessed on a Likert scale (1–5, ranging from rarely to very frequent). Participants who reported changes or difficulties were asked to describe them.
- Behaviors presented by the person with MND – 12 questions. Changes in behavior, mood, adaptation to the routine, and adherence to protective measures were evaluated on a Likert scale (1–5, ranging from improvement in mood/behavior to worsening of mood/behavior, or difficulties in adapting to the routine and adhering to protective measures). Participants who reported changes or difficulties were asked to describe them.

Three professionals who work with caregivers of people with MND evaluated the first version of the interview. After the inclusion of suggestions from these professionals, two caregivers who were not part of the final sample were interviewed to verify adequacy of the questions and inclusion of other suggestions.

#### Self-Reporting Questionnaire (SRQ-20)

The World Health Organization developed the SRQ-20^14,15^ to screen for symptoms of nonpsychotic mental disorders for the general population.^14,16^ The Brazilian version of SRQ-20^17,18^ was validated to the Brazilian population in different settings.^15^ SRQ-20 consists of 20 dichotomous questions (yes/no). Its score ranges from 0 to 20, and the cutoff point used is 7/8, based on Brazilian studies to discriminate cases in which there are emotional disorders. After each SRQ-20 question, the participants were asked to assess whether their current status concerning the topic covered was better, worse, or the same compared to the period before the quarantine.

### Procedures

Potential participants were contacted by phone, via text message, explaining the study aims and inviting them to participate. People who responded positively were contacted later via voice call to answer questions and schedule the interview. Before starting the interview, the general terms of participation in the study were read to the participant. Their oral consent was requested, recorded using the “Call Recorder” application for android phones. A copy of the document was sent to the participant via cell phone. During the interview, which lasted approximately 20 minutes, the researcher asked the questions and recorded the participant’s answers on a form. Each interview was audio-recorded to facilitate later confirmation that participant responses were accurately documented. At the end of the interviews, the researcher thanked the participants for their participation and sent them the written material produced by the Brazilian Alzheimer’s Association (ABRAz) with useful, specific information about COVID-19 for people with MND. The study was approved by the Brazilian ethical review bodies (National Council on Ethics in Human Research — CONEP) before its realization (CAAE 89585318.1.0000.5407 authorization).

### Data analysis

The data from the variables related to the caregiver’s characteristics, the person with MND, changes in routine due to quarantine, and the classification of the SRQ-20 instrument (<8 — no-risk; >8 — risk) were assessed using descriptive statistics. The first three groups of variables were compared considering the two groups defined by the categorization of the SRQ-20 instrument, and the quantitative variables were assessed for normality (Shapiro-Wilk test) and homoscedasticity (F test) and analyzed using the Student’s *t*-test test for independent samples when these assumptions were accepted or using the Mann-Whitney U nonparametric test when rejected. Qualitative variables were analyzed using the chi-square test for independence, followed by the adjusted residuals test in statistical significance cases. In all inference tests, a p-value of 0.5 was considered significant.

Variance partitioning analysis was also performed, testing the effects of the different matrices of independent variables (x-axis — characteristics of the caregiver, characteristics of the person with MND, change in the routine) on the values of the SRQ-20 dependent variable (y-axis). Partial distance-based redundancy analysis (dbRDA) was used to test each matrix of independent variables’ effect, and the results were interpreted together. The partial dbRDA was calculated using the “rda” function with the “Bray-Curtis” distance of the vegan package. All analyses were performed using the R software, version 3.6.0 (R Development Core Team 2018), considering a significance of p<0.05.

## RESULTS

### Characteristics of the caregiver

We collected data between April and May 2020, a month after the quarantine in Brazil was announced. All participants were women. The sample was predominantly composed of married women (65.7%), daughters (62.8%) of the person they cared for, without formal work (51.4% — homemakers, retired, or unemployed), with a family income of up to about US$800.00 per month (65.7%; however, 37.1% had a monthly income of up to about US$400.00). Approximately 74.0% had some help from a family member to care for the person with dementia. Thirty (85.7%) participants stated that they were very or extremely frightened (level 4 or 5, on a scale of 0–5) that the person with MND would get COVID-19, and 40.0% of the sample said they felt the same intensity of fear regarding their own contamination.

Eleven (31.4%) participants had a score >8 on the SRQ-20, which characterizes risks to these participants’ mental health. Item analysis of SRQ-20 demonstrated that 80% of the participants reported worsening in at least 1 item of the questionnaire, 48.6% reported worsening in 1–5 items, 20.0% worsening in 6–10 items, and 11.4% reported worsening in 11 or more. The symptoms that worsened during the quarantine reported by the participants were nervousness (51.4%), sadness (37.1%), and sleep (34.3%). However, 31.4% of the participants (n=11) reported feeling better about at least one of the items evaluated: nine of them reported improvement in 1–5 items and another two in more than 6 items. The symptoms that the participants most perceived as having improved were tiredness (14.3%) and crying (11.4%).

The presence or absence of risk for mental health (score >8 or <8 in the SRQ-20, respectively) was analyzed with other variables, such as characteristics of caregivers and persons with MND and routine changes. When analyzing the variables related to the caregiver’s characteristics, it was possible to verify that no single variable showed statistically significant differences between the groups no-risk and risk for mental health (Table 1). The scores obtained in the SRQ-20 instrument effectively separated the two groups (no-risk and risk) significantly (p<0.0001).

**Table 1.** Variables relating to the characteristics of the caregiver to the whole group and according to the risk classification defined by the SRQ-20 instrument (No-Risk and Risk for mental health).

		Whole group (n=35)		No-risk (n=24)		Risk (n=11)		p-value
Age (years), mean±SD		52.2±10.1		52.5±7.5		51.5±15.4		0.855
Care time (months), mean±SD		42.1±35.4		42.3±35.5		41.6±38.6		0.829
SRQ-20		6.5±4.51		4.00±2.45		12±3.07		<0.0001
Marital status, n (%)	Single	6	17.1	3	12.5	3	27.3	0.580
	Married	23	65.7	16	66.7	7	63.6	
	Divorced	4	11.4	3	12.5	1	9.1	
	Widowed	2	5.7	2	8.3	0	.00	
Formal work, n (%)	No	18	51.4	14	58.3	4	36.4	0.399
	Yes	17	48.6	10	41.7	7	63.6	
Education, n (%)	1	1	2.8	1	4.2	0	.00	0.920
	2	6	17.1	4	16.7	2	18.2	
	3	13	37.1	9	37.5	4	36.4	
	4	15	42.8	10	41.7	5	45.4	
Income, n (%)	1–2 MW	13	37.1	10	41.7	3	27.3	0.854
	2–4 MW	10	28.6	6	25.0	4	36.4	
	4–6 MW	9	25.7	6	25.0	3	27.3	
	+6 MW	3	8.6	2	8.3	1	9.1	
Care help, n (%)	No	9	25.7	5	20.8	4	36.4	0.576
	Yes	26	74.3	19	79.2	7	63.6	
Care of >1 person	No	26	74.3	19	79.2	7	63.6	0.576
	Yes	9	25.7	5	20.8	4	36.4	
Fear of contamination of oneself, n (%)	1	7	20.0	5	20.8	2	18.2	0.209
	2	11	31.4	9	37.5	2	18.2	
	3	3	8.6	3	12.5	0	0.0	
	4	14	40.0	7	29.2	7	63.6	
	5	0	0.0	0	0.0	0	0.0	
Fear of contamination of the care receiver, n (%)	1	1	2.8	1	4.2	0	0.0	0.552
	2	1	2.8	1	4.2	0	0.0	
	3	3	8.6	3	12.5	0	0.0	
	4	21	60.0	14	58.3	7	63.6	
	5	9	25.7	5	20.8	4	36.4	

MW: monthly minimum wage (1 MW=~US$209.00). p-value of significance tests.

### Characteristics of the people with major neurocognitive disorder

The people with MND were mostly women (60.0%), with a mean age of 82.6 years and a diagnosis of Alzheimer’s disease (65.7%) between 4 months to 15 years. Almost half of them (48.6%) were bedridden (n=5; 14.2%) or did not usually leave home before the pandemic (n=10; 42.8%). The diagnosis of MND was ascertained from the caregiver’s report. Most of the caregivers did not notice changes in the care receivers’ behavior or mood, except in the Risk group, in which almost half of the sample noticed some changes in the person’s mood. Most caregivers considered that persons with MND were fully adapted to the quarantine changes (level 1, on a scale of 1–5). When evaluating the variables related to the person with MND, no single variable showed statistically significant differences between the groups no-risk and risk for mental health (Table 2).

**Table 2.** Variables relating to the characteristics of the person with MND to the whole group and according to the risk classification defined by the SRQ-20 instrument (no-risk and risk for mental health).

	Level	Whole croup (n=35)		No-risk (n=24)		Risk (n=11)		p-value
Age (years), mean±SD		82.5±7.07		82.4±8.11		81.8±4.8		0.835
Change in behavior, n (%)	1	0	0.0	0	0.0	0	0.0	0.168
	2	4	11.4	4	16.7	0	0.0	
	3	17	48.6	11	45.8	6	54.5	
	4	7	20	6	25.0	1	9.1	
	5	7	20	3	12.5	4	36.4	
Change in mood, n (%)	1	1	2.8	1	4.2	0	0.0	0.391
	2	2	5.7	2	8.3	0	0.0	
	3	19	54.3	14	58.3	5	45.4	
	4	9	25.7	4	16.7	5	45.4	
	5	4	11.4	3	12.5	1	9.1	
Adaptation to the quarantine, n (%)	1	18	51.4	13	54.2	5	45.4	0.8731
	2	4	11.4	2	8.3	2	18.2	
	3	9	25.7	6	25.0	3	27.3	
	4	1	2.8	1	4.2	0	0.0	
	5	3	8.6	2	8.3	1	9.1	
Bedridden or not leaving home, n (%)	No	18	51.4	10	41.7	8	72.7	0.179
	Yes	17	48.6	14	58.3	3	27.3	

Changes in behavior and changes in mood: 1=much better; 2=a little better; 3=has not changed; 4=a little worse; 5=much worse; Adaptation to the quarantine: 1=fully adapted; 2=very adapted; 3=partially adapted; 4=poorly adapted; 5=did not adapt.

About 40.0% of caregivers reported an increase in challenging behaviors in their family member with dementia, mainly agitation, aggression, and confusion. Among those who reported worsening mood (31.7%), the symptoms of irritation, anxiety, sadness, and apathy stood out. Caregivers attributed these symptoms to the impossibility of the person leaving home and to the lack of contact with family members. Difficulty in adapting the care receiver to the new routine (28.0%) was attributed to the fact that the people with MND had stopped attending consultations (e.g., physiotherapy, psychology, occupational therapy, and health centers) and had to maintain social distance from friends and family.

### Changes in the routine

No single variable showed statistically significant differences between the no-risk and risk for mental health groups (Table 3), except for a tendency toward difficulty in the care (p=0.055). Most of the Risk group (72.7%) reported having great difficulty caring for the person during the pandemic. In comparison, 45.0% of no-risk group reported no difficulty in care in the pandemic. However, all participants recognized that the quarantine caused changes in the routine, especially in demand for additional care family member with MND.

**Table 3.** Variables relating to the change of routine to the whole group and according to the risk classification defined by the SRQ-20 instrument (No-Risk and Risk for mental health).

	Level	Whole group (n=35)		No-risk (n=24)		Risk (n=11)		p-value
General change, n (%)	1	0	0.0	0	0.0	0	0.0	0.084
	2	6	17.1	6	25.0	0	0.0	
	3	8	22.8	7	29.2	1	9.1	
	4	15	42.8	8	33.3	7	63.6	
	5	6	17.1	3	12.5	3	27.3	
Change in the work, n (%)	1	7	20.0	4	16.7	3	27.3	0.472
	2	5	14.3	4	16.7	1	9.1	
	3	3	8.6	2	8.3	1	9.1	
	4	15	42.8	9	37.5	6	54.5	
	5	5	14.3	5	20.8	0	0.0	
Change in the family member care, n (%)	1	3	8.6	3	12.5	0	0.0	0.447
	2	5	14.3	4	16.7	1	9.1	
	3	6	17.1	5	20.8	1	9.1	
	4	18	51.4	10	41.7	8	72.7	
	5	3	8.6	2	8.3	1	9.1	
Difficulty in the family member care, n (%)	1	12	34.3	11	45.8	1	9.1	0.055
	2	3	8.6	3	12.5	0	0.0	
	3	5	14.3	3	12.5	2	18.2	
	4	14	40.0	6	25.0	8	72.7	
	5	1	2.8	1	4.2	0	0.0	

p-value of significance tests.

Approximately 60.0% of the participants noticed very significant changes in their routine (levels 4 and 5), and 91.4% reported at least one critical change in their general routine. The most frequently mentioned changes were alterations in hygiene routines, the impossibility of leaving home or receiving visits, increased care time with the care receiver, and the lack of external help for the care. Approximately 63.0% of the total sample cited at least one difficulty caring for the person with MND. The most cited were dealing with changes in the person’s mood or behavior, maintaining social distancing, and taking care of all the new tasks to be performed with them.

### Variance partitioning of the Self-Reporting Questionnaire values in relation to the predictor variables

**Figure 2.**
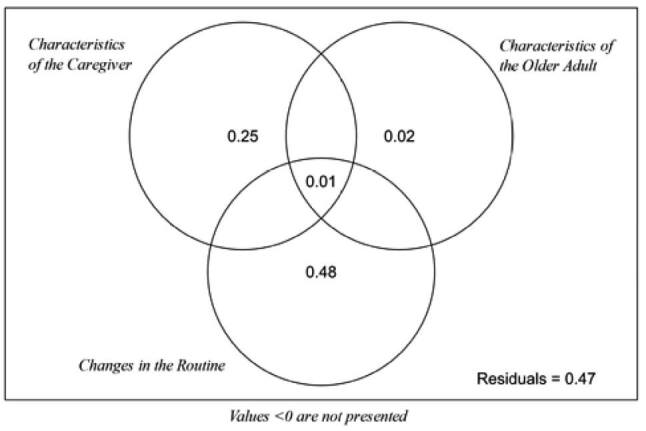
Variance partitioning of the matrix of characteristics of the caregiver, of the care receiver and changes in the routine, with exhibition of the respective percentages of unique and shared explanation.

In the variance partitioning analysis, variables related to the caregiver’s characteristics and those related to changes in the routine significantly affected the SRQ-20 scores (F=3.408, p=0.024; F=6.556, p=0.002, respectively). The variables related to the older adult did not influence the variation of the scores (F=0.907, p=0.498; Figure 2). The data matrices related to the caregiver variables’ characteristics affected 25.0% of the variation of the SRQ-20 scores, while the changes in the routine variables affected 48.0%. The characteristics related to the care receiver affected only 2.0% of the variations in the SRQ-20 scores, not being considered a significant contribution (p>0.05).

## DISCUSSION

Caregivers of people with MND are more prone to stress and burden.^19^ In situations of social distancing, they are supposed to be at higher risk of developing mental disorders,^20^ since, in the daily care, they usually report feelings of loneliness, isolation, and lack of social support.^21,22^ However, there is little literature about this topic, especially during imposed social isolation measures, such as those that occurred during the COVID-19 pandemic, which justified the aim of this study. We found a high prevalence of indicators of mental health problems in the studied sample (in a population-based survey^23^ conducted in the capital of southern Brazil in 2017, e.g., the prevalence of common mental disorders was 14.7%). However, these results are similar to the indicators of mental health problems found in the general population during the quarantine period. In a study conducted in China with the general population during the quarantine, 33.2% of individuals had scores above the cutoff point in the SRQ-20.^24^ A systematic review and meta-analysis of studies on the rates of stress, anxiety, and depression in the general population during the pandemic found a prevalence of 29.6, 31.9, and 33.7%, respectively.^25^ It is possible to assume, therefore, that the quarantine did not have a greater impact on the sample of caregivers of people with dementia studied than on the general population, as found in studies conducted in other countries.

Besides, the rate of indicators of mental health problems found among the participants in this study does not differ from that found in other studies conducted with caregivers of people with dementia during periods outside the COVID-19 pandemic in Brazil (32.3%)^26^ and in Latin American countries (e.g., 30.5% in the Dominican Republic).^27^ Similar levels of mental health problems found among this group of caregivers of people with dementia before and during the quarantine period do not necessarily mean that these individuals were not affected by the social distancing measures. This study group cannot be generalized because it comprises a small group of caregivers.

It should be emphasized that the care situations of people with dementia have been correlated with high rates of mental health problems in caregivers.^28^ In the current study, most of the participants reported that some of the symptoms investigated worsened, especially nervousness, sadness, and sleep. The combined results of this study could suggest that, instead of increasing the number of indicators of mental health problems, the quarantine may have resulted in a worsening of preexisting symptoms. Social isolation may have aggravated the difficulties previously faced by these caregivers, affecting the more vulnerable individuals in the sample and those who were already weakened due to the situation of long-term care for their family member with dementia. However, this hypothesis should be viewed with caution since we do not have realistic estimates of how the sample participants were before the pandemic.

It was evident that caregivers identified as being in the risk group noticed more significant difficulties in caring for the people with MND than the no-risk group. The quarantine imposed rigid hygiene routines, impossibility to leave home or receive visits, increased care time with the care receiver, and limited access to external help for the care, which may have added stress to the difficulties faced before the quarantine. These factors, combined with the caregiver’s characteristics, were the variables that most affected the score of the participants in the SRQ-20. However, some participants of the no-risk group (but none of the risk group) reported improved symptoms investigated in the SRQ-20, mostly tiredness. Considering that most of the participants carried out formal work and the caregiver activity, the reduction of working hours or telecommuting in the quarantine may have contributed to some participants feeling less tired or perceiving improvements in some of the symptoms investigated. It is also essential to consider that, for some families, the absence of constant visits from other family members can prevent preexisting conflicts, as reported by at least two participants. The improvement in some symptoms investigated (but not necessarily at all) indicates that quarantine’s effects should be analyzed according to the caregivers’ social, economic, and family conditions.

The majority of the caregivers did not notice changes in the care recipients’ mood or behavior, reporting that they had adapted to the quarantine changes. This result may mean that the changes resulting from the quarantine can more significantly affect people with MND. They have some vulnerabilities that cannot be identified in the present study. A similar result was found in a study conducted in France^29^ with 38 caregivers of people with MND. Only 10 (26%) caregivers related neuropsychiatric changes in care recipients during the quarantine. The severity of these symptoms was significantly correlated with both the duration of social isolation and lower cognitive function prior quarantine period. Future studies should investigate factors that may predispose people with MND to present changes in mood and behavior problems.

The small sample size constitutes a limitation of this study due to difficulty contacting participants during the quarantine. The recruitment of the sample took place from a limited list of potential participants and the rate response of about 36% may have caused a sampling bias. As the participants’ recruitment was carried out at the beginning of the quarantine in Brazil, it is possible that people with more adaptation difficulties refused to participate in the research. In addition, many informal caregivers of older adults have difficulties to access online forms (e.g., low income, no access to the Internet and electronic devices), which meant that the participants’ approach occurred via telephone and not by sending forms. However, although the group was small, it represented the population of caregivers of people with MND.^30,31^

A sample consisted of women only should also be considered a limitation of the study. Despite global estimates for low- and middle-income countries^32^ indicating that women are primarily responsible for the informal care of people with MND,^19^ women seem to be more vulnerable to disorders such as depression and stress in general,^33,34^ as confirmed in a study conducted during the COVID-19 pandemic.^24^ Therefore, a sample consisting only of women may represent a bias in the prevalence of indicators of mental health problems found in the current study. Another limitation is that we did not measure mental health indicators in the sample before the pandemic period. Therefore, we cannot assess the evolution of the symptoms. Also, it is necessary to consider that 48% of the sample of people with MND cared for was bedridden or did not leave home before the pandemic, which usually represents a more severe stage of the disorder. Although this characteristic does not seem to have contributed to the presence or absence of mental disorders indicators, it constitutes a limitation of this study.

This study’s strength is to evaluate caregivers of persons with MND 1 month after the announcement of quarantine in Brazil. The study participants’ recruitment took place immediately after COVID-19 pandemic (first 3 months). Therefore, we assessed this vulnerable cohort in a unique scenario. Concomitantly, it constitutes a limitation of the study since we did not have the time or opportunities to select the study group. Further studies should then include a more heterogeneous group of caregivers and care receivers with MND, allowing more external validity of these results. It would also be essential to assess the effects of quarantine on this population in the longer term to verify whether the restriction measures’ continuity could change the situation observed in the present study.

To summarize, there were high indices of mental disorders in the studied sample. However, these high indicators also did not differ from the prevalence of mental health problems in the general population during COVID-19 pandemic or caregivers before it. These arguments tend to support the hypothesis that quarantine may have worsened preexisting symptoms in caregivers of persons with MND. Also, it highlights that social isolation is already a reality for caregivers and family member with MND that do not find support in the community or health care facilities. Informal care is a rule in our country, and the prohibition of family visits was the main restriction that social isolation imposed for these participants. Caregivers and care receivers’ characteristics may explain the results presented since high mental health indicators could be attributed to changes in the routine. Feeling adapted completely to changes could be related to the care receivers’ characteristics and caregiver burden before COVID-19. However, future studies should clarify the causes of these findings.

Results presented here show the complexity of this topic and the need for individual care for this group, especially in situations like the COVID-19 pandemic. Guidance on how to deal with changes in the routine related to patient care^35^ could reduce the stress resulting from the social isolation measures. Online support groups for caregivers may also help them understand the sources of stress and suffering and encourage dialogue and sharing experiences during the pandemic.^20^
